# Meeting Review on India EMBO lecture course on RNA-protein complexes: from molecular assembly to physiological functions and disease

**DOI:** 10.1242/bio.062148

**Published:** 2026-01-26

**Authors:** Debleena Mukhopadhyay, Nasrin Banu Mohammad Faisal, Chloe Leray, Sania Sultana

**Affiliations:** ^1^ National Centre for Cell Science, University of Pune Campus, Pune University Road, Ganeshkhind, Pune, Maharashtra 411007, India; ^2^ Université Côte d'Azur, CNRS, Inserm, Institut de Biologie Valrose, 06108 Nice cedex 2, France; ^3^ National Brain Research Centre, Manesar, Gurugram, Haryana 122052, India

**Keywords:** India EMBO lecture course, Meeting report, RBPs, RNA and RBPs

## Abstract

The India EMBO lecture course ‘RNA-protein complexes: from molecular assembly to physiological functions and disease’ was held at The National Centre for Cell Science, Pune, India, from February 24 to 28, 2025. The major theme of the lecture series centred on the recent advances in RNA-protein interactions and their role in regulating complex assembly or condensation as well as cellular functions and plasticity. Additionally, the course highlighted the impact of dysregulated post-transcriptional processes in various diseases. Speakers from various biological disciplines presented their research on both the fundamental architecture of RNA and protein complexes and their contributions to higher-order cellular functions. The course also featured flash talks and poster presentations selected from abstract submissions, alongside special methodological workshops on omics and phase separation. This Meeting Review reflects on the event's key discussions, drawing attention to the overarching themes and main conclusions.

## Teaser

**Summary:** This Meeting Review highlights talks from some of the renowned labs working on RNA and RBPs who participated in the EMBO lecture course ‘RNA-protein complexes: from molecular assembly to physiological functions and disease’ and gives an insight into their current work.

## Introduction

RNA–protein interactions are crucial for most biological processes, playing a key role in gene regulation and cellular homeostasis (Meyer, 2018; Henninger et al., 2021). Aberrant RNA-RNA binding protein (RBP) interactions have been implicated in various diseases, including neurodegenerative diseases (Cid-Samper et al., 2018) and cancers (Wang et al., 2024). Recent advances in our understanding of RBPs-RNA interactions have provided insights into their dynamic assembly into nuclear or cytoplasmic higher-order ribonucleoprotein (RNP) complexes, whose biological functions have started to emerge (Rodgers and Woodson, 2021).

**Fig. 1.**
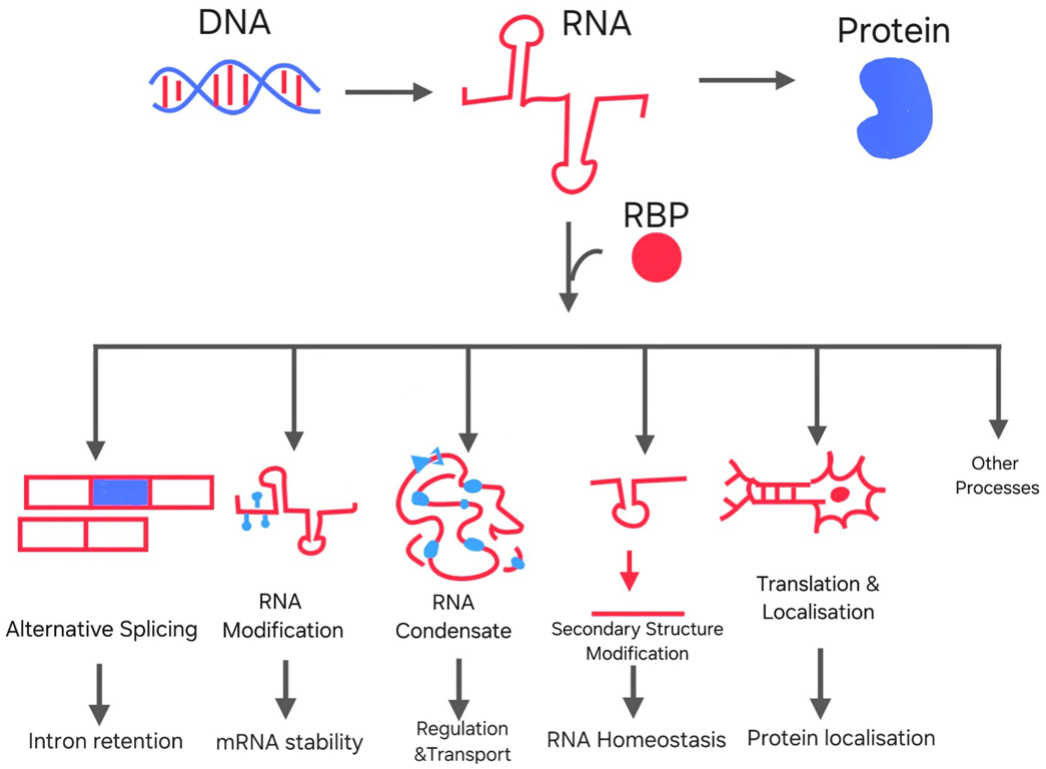
**Schematic diagram representing the topics of the conference, illustrated by different examples.** DNA is transcribed into RNA, which is then translated to the respective proteins. RNA can also interact with other RBPs and take part in various types of mechanisms (as mentioned in the diagram), which are ultimately responsible for several important cellular processes like mRNA stability, homeostasis, protein localization, etc.

RNA in the nucleus is produced through the process of transcription and undergoes several processes and modifications such as splicing (Wang et al., 2014), and chromatin-associated regulation (Patrasso et al., 2023) (Fig. 1). In the cytoplasm, mature RNAs participate in translation or are localised to specific subcellular locations, such as neuronal synapses (Bourke et al., 2023) or oocyte poles essential for embryonic development (Medioni et al., 2012), to streamline protein synthesis in a spatiotemporal fashion. Furthermore, RBPs engage in multivalent interactions, enabling the formation of phase-separated compartments, like stress granules (Molliex et al., 2015) and P-bodies (Luo et al., 2018). Disturbances in these highly regulated processes can lead to abnormal RNPs, which may contribute to the development of diseases.

Recent technological breakthroughs have greatly advanced our ability to explore RNA–protein interactions at both the molecular and systems levels. These include high-throughput RNA structure probing (Piao et al., 2017), advanced interactomics (Schönberger et al., 2018) and live-cell RNA imaging (Pham and Wu, 2024), and biomolecular condensation assays (Linsenmeier et al., 2021). Together, these tools have transformed our understanding of RNA metabolism and RNP dynamics in health and disease.

The India EMBO lecture course, ‘RNA–protein complexes: from molecular assembly to physiological functions and disease’, introduced participants to these emerging concepts and methodologies. The overarching themes of the course focused on the molecular mechanisms underlying RBP, RNA and omics; RBP, RNA in neurons; RNA condensates; RBP and RNA structure; RBP and RNA imaging; and RBP and RNA in cellular physiology, disease and development. A total of 193 attendees participated in the lecture course. 23 speakers from different fields of biology were invited to share their work on RNA-protein interaction, 43% of whom were female. In addition to the talks, specialised workshops were organised on imaging, phase separation and omics to help students better understand the new methodologies to explore the dynamic interactions of RNA-proteins. The course not only included talks but also provided students with opportunities to engage with the speakers through journal clubs and informal discussions, offering a stimulating collaborative learning environment.

## RBP, RNA and omics

The opening plenary lecture delivered by Matthias Hentze (EMBL, Germany) explained the concept of riboregulation, in which RNA binding regulates the activity of proteins. This concept emerged from a technique developed in his lab, the RNA interaction capture (RIC), used to systematically discover proteins binding to RNA (Castello et al., 2012). He described mechanisms of riboregulation with two examples: one was the riboregulation of ENO1 in mESC differentiation (Huppertz et al., 2022), and the other was the riboregulation mechanism in controlling selective autophagy by p62, a non-classical RNA-binding protein (Horos et al., 2019; Büscher et al., 2022).

Kathi Zarnack (University of Würzburg, Germany) talked about the predominant chemical modification of RNA called N6-methyladenosine, which plays a critical role in mRNA stability (Zhou et al., 2024). Her group found that m6A sites in coding regions destabilizes the RNA through a novel pathway, CMD (CDS-m6A decay), which relies on ribosomal pausing leading to RNA destabilization. Interestingly, the targets of CMD are mainly localized in processing bodies (P-bodies). Additionally, CMD safeguards the genome against retrogenes, which generally got upregulated when CMD was inhibited.

Joel Perez-Perri (iMM, Portugal) introduced a new method called enhanced RNA interactome capture (Perez-Perri et al., 2021), which overcomes technical limitations of RIC, enabling identification of RNA-interacting proteins (e.g. m6A responsive RBPs) from cultured cell and mammalian organs (Perez-Perri et al., 2023) with greater specificity and increased signal-to-noise ratio.

The last talk of the session was an EMBO Global lecture series given by Maria Carmo Fonseca (iMM, Portugal). Her talk described the discovery of PCATs, which stands for promoter proximal convergent antisense transcripts. Using POINT (polymerase associated intact nascent transcript) technology (Sousa-Luís et al., 2021), they found that PCATs are found near the transcription start site of 40% of transcribed human protein coding genes. They also found that the majority of the genes which get activated during iPSC differentiation are associated with PCATs. PCATs initiate upstream of paused polymerase II via formation of R-loops. Promoters that are associated with PCATs have been found to have extended CG-rich sequences and PCATs possibly enhance the transcriptional activation by keeping the chromatin open around the TSS.

Ted Abel (Carver College of Medicine, USA) discussed the molecular and circuit-level mechanisms that underlie autism spectrum disorder (ASD). Using 16p11.2 deletion mouse models (16p11.2 del/+), he demonstrated male-specific abnormalities linked to striatal dysfunction in ASD. He highlighted how impairments in reward learning and motivation are primarily mediated by the dopamine receptor pathway (Kim et al., 2024).

Overall, the talks highlighted emerging insights into RNA regulation and local translation in neurons, particularly underlying learning and memory. Additionally, the presentations also emphasised sex as an important biological variable in studying ASD.

## RBP, RNA in neurons

Neurons have evolved specialised mechanisms for regulating RNA metabolism, which have been reported in complex cellular functions, such as neuronal development, plasticity, and pathophysiology of various neurological disorders (Lemmens et al., 2010; Du et al., 2019). The meeting presented recent advances in the understanding of transcriptional and translational regulation in neurons.

The session opened with Oriane Mauger from the Max Planck Institute of Psychiatry, Germany, discussing transcripts retained in the nucleus, which are mostly stable intron-retaining mRNAs that have widespread roles, including degradation, splicing, and stimulus-dependent localisation to the cytosol (Mazille et al., 2022).

Kathryn Meyer (Duke University School of Medicine, USA) provided key insights into m⁶A enrichment at 3′UTRs and near stop codons with GAC and AAC motifs. Using MeRIP-seq, her team also contributed to the first transcriptome-wide profile of m⁶A (Meyer et al., 2012). She also introduced single-cell DART-seq (scDART-seq), which allows single-cell m⁶A mapping, revealing heterogeneity across individual cells (Tegowski et al., 2022).

Valérie Hilgers (MPI of Immunobiology, Germany) highlighted the differences in RNA expression between neurons and other cell types. She demonstrated that extended neuronal 3′ UTRs are found exclusively in cells that express ELAV, a conserved neuron-specific RNA-binding protein (Castro Alvarez et al., 2021). ELAV also serves as a master regulator of neuronal exons and circular RNA, which are produced during back splicing (Alfonso-Gonzalez et al., 2025).

Eric Klann (NYU, USA) presented evidence regarding the role of eukaryotic initiation factors (eIFs), specifically eIF2α (Sharma et al., 2023) and eIF4E-dependent translation (Gindina et al., 2021) in memory consolidation following fear conditioning. He also introduced the development of iPKR knock-in/knock-out mouse lines, featuring cell-type-specific drug-inducible protein synthesis inhibition (ciPSI). These transgenic mice enable the controlled phosphorylation of eIF2α, leading to translational inhibition, thereby providing a powerful tool to probe into the translation-dependent mechanism of memory formation (Sharma et al., 2020).

## RNA condensates

RNA condensation occurs under a variety of physiological and pathological conditions, including quiescence, development, and disease (Wadsworth et al., 2024; Coulon and Vaurs, 2020; Bose et al., 2022). However, the mechanisms underlying the formation of these condensates and their functional roles remain poorly understood. Importantly, recent findings suggest that some observed condensates may be artefacts caused by protein tagging, highlighting the need for careful experimental design (Avecilla et al., 2025).

The session began with Arnaud Hubstenberger (iBV, France), who presented evidence that RNA condensates possess distinct physical properties, which can be altered by mutations in the RNA helicase CGH-1 (Hubstenberger et al., 2015). He also described how, during quiescence, homotypic RNA nanoclusters assemble into larger macro-condensates (Cardona et al., 2023), suggesting a regulated, multiscale process of condensate organization.

Next, Mainak Bose (IIT Kharagpur, India) discussed the functional role of *oskar* RNP granules in *Drosophila* oocytes. He demonstrated that these granules mature into a solid-like state, a process that is essential for efficient RNA transport and posterior localization and local translation of *oskar* mRNA during oocyte development (Bose et al., 2022).

Finally, Maria Hondele (University of Basel, Switzerland) provided critical insights into potential artefacts introduced by protein tagging when studying condensate behaviour. She showed that different tags can dramatically alter the phase behaviour of tagged proteins, potentially leading to misleading conclusions (Dörner et al., 2026).

These presentations collectively emphasized the complexity and diversity of RNA condensates across biological contexts. While significant progress has been made in identifying their biophysical properties and biological functions, important challenges remain – particularly in distinguishing physiological condensates from experimental artefacts.

## RBP and RNA structure

RBPs and RNA structure, have a central role in gene expression regulation and disease. Varun Bhaskar (JNCASR, India) presented work on how FMR1 and the C9orf72–SMCR8 complex influence translation and RNA homeostasis, particularly in the context of ALS/FTD (Luteijn et al., 2025). Sutapa Chakrabarti (Freie Universität Berlin, Germany) detailed the multifunctional activity of UPF1 across nonsense-mediated decay (NMD), Staufen-mediated decay (SMD), and histone mRNA decay (HMD), highlighting the regulatory role of co-factors such as Stau1 and SLBP (Gowravaram et al., 2019). Kevin Weeks (University of North Carolina at Chapel Hill, USA) introduced a novel chemical probing approach to map RNA tertiary structures using TMO, identifying critical structural sites (T-sites) that influence gene expression, as shown in the CTC1 RNA. Christine Mayr (Sloan Kettering Institution, USA) concluded the session by demonstrating how the 3′ UTR region of mRNAs can govern protein localization and activity, particularly for large proteins with intrinsically disordered regions, exemplified by KDM6B/JMJD3. Collectively, these talks highlighted how structural and mechanistic studies of RNPs are reshaping our understanding of RNA regulation in both physiological and pathological contexts.

## RBP and RNA imaging

One of the important aspects of understanding the role of RBP and RNA interaction is studying the interactions *in vivo* and *in vitro*. With the development of fluorescence reporters and quantitative imaging in live and fixed cells or tissues, RBP and RNA interactions and the dynamics of such interactions can be studied in much detail (Xia et al., 2017; Rath and Rentmeister, 2015).

Jeffrey Chao (Friedrich Miescher Institute, Switzerland) introduced an imaging tool, TREAT (3′ RNA end accumulation during turnover), which they used to identify translation-dependent regulation of mRNA stability, a process that depends on ribosome flux (Dave et al., 2023). Using alternative single molecule labelling and imaging techniques, they elucidated the role of LARP1 and 4EBP1/2 on 5′ TOP mRNA in context of mTOR signalling (Hochstoeger et al., 2024) and studied translation occurring within stress granules (Mateju et al., 2020).

Next, Edouard Bertrand (IGH, France) described how localization of some RNAs is a translation dependent process and how the nascent protein can drive the polysome to its targeted site. His group elucidated this mechanism of ‘polysome sorting’ by studying how the interaction of β-catenin mRNA with either APC or E-cadherin can drive differential subcellular localization and regulation (Salloum et al., 2024 preprint). Together, these talks emphasized how advanced imaging tools and techniques can be used to study RBPs and RNA dynamics with high spatio-temporal resolution and precisely understand their role and regulation.

## RBP and RNA in cellular physiology, disease and development

One of the major aspects of studying RBP and RNA is to understand their various roles in the context of diseases, development and cellular physiology. Recent studies have focused on what the role of these RBPs is, other than the well-known general mechanism of transcription and translation (Gebauer et al., 2021; Li et al., 2024; Fabbiano et al., 2020).

Dasaradhi Palakodeti (Instem, India) explained the role of RNP granules in mediating regulation of mitochondrial states, which is necessary for maintaining pluripotency (Mohamed Haroon et al., 2021), specifically TDRD9, which is important for stem cell regeneration and, mitochondrial localization and cell cycle phase transition in neoblasts (D. Palakodeti, unpublished observations/data).

Martin Turner (Babraham Institute, UK) talked about how lymphocyte cell fate and immunity can be determined by RNA binding proteins – specifically serine/arginine-rich splicing factors (M. Turner, unpublished observations/data) and ELAVL1, which have a role in proliferation of B-cells (Turner and Díaz-Muñoz, 2018), and Tristetrapolin-ZFP36, which has a role in regulating thymic development, the deletion of which can cause T lymphoblastic leukaemia (Hodson et al., 2010), while in mature T cells these function to limit immune responses (Petkau et al., 2022; Sáenz-Narciso et al., 2025).

Sonam Dhamija (IGIB, India) explained how non-coding sequence can become coding and explained the relevance of non-stop extension mutation (NSExt^mut^) in cancer specifically in context of SMAD4, VHL and BAP1 expression (Dhamija et al., 2020; Pal et al., 2025).

Javier Martinez (Max Peutz Lab, Austria) talked about the human tRNA ligase complex (tRNA-LC) and how they found that all paralogs of FAM98 are assembled into the tRNA-LC. Additionally, they studied the role of Ashwin in the context of tRNA-LC function and localization (Kroupova et al., 2021).

Chandrama Mukherjee (Presidency University, India) explained how recapping of cytoplasmic RNA after cellular stress takes place by formation of cytoplasmic capping complex (CCE) and elucidated about the shuttling of CCE between cytoplasm and nucleus and how CCE associates with stress granules, while also briefly mentioning the role of lncRNA in context of CCE (Gayen et al., 2024; Mukherjee et al., 2023).

Overall, this session focused on the functions of RBPs in a broader context and gave a glimpse into the various cellular roles that are dependent on RBPs.

## Conclusion

**Fig. 2.**
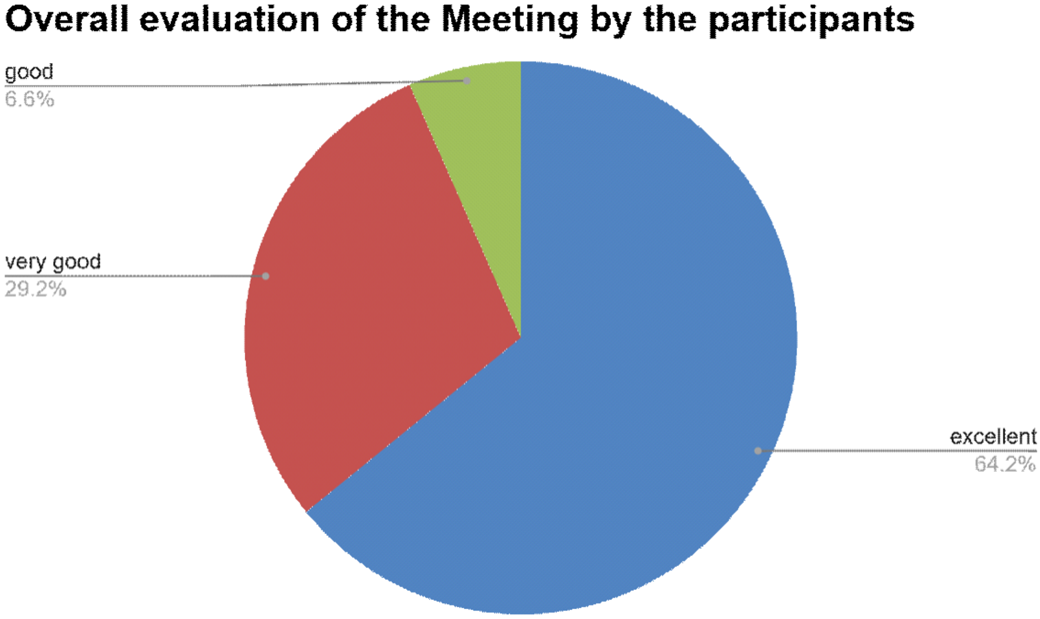
**Pie chart depicting the overall evaluation of the meeting by the participants.** Data were collected through a questionnaire by the EMBO organizers.

This EMBO lecture course provided an excellent platform for sharing cutting-edge knowledge on RNA–protein complexes, while also equipping participants with a wide range of experimental and analytical tools to study them. This sentiment was widely shared among attendees: 100% felt that the course adequately covered the topic, and 97% rated the quality of the talks as very good or excellent (Fig. 2) and the course is proposed to be conducted again in 2028.

Beyond the scientific content, the course also fostered collaboration and networking through its non-traditional session formats, including technological workshops and journal clubs. These formats were highly appreciated, with 96% of participants reporting that they had sufficient or ample time to interact and connect with other attendees.

Some of the feedback received by the EMBO organizers from a participants’ questionnaire regarding the meeting is included below:

“Excellent program, should organize again.”

“This meeting was very beautifully organised by the organisers without any glitches. Especially I would like to extend my thank you to the student volunteers involved, they worked relentlessly throughout and made this a success.”

“It was an excellent line up of speakers and also at different stages in the career which really helped every single participant. The arrangement of the sessions was also very good and well thought, since it provided good networking opportunities and discussion time.”

“Excellent organisation and management by the full team of NCCS. Scientific interactions were really very useful.”

“The organization of the course was excellent. Including accommodation in the registration fee would make it more convenient.”

“It was a very well organized meeting, with excellent speakers and ample of opportunities to communicate.”

“It was an excellent course, far exceeding the quality of similar meetings I have attended in Europe. The quality of the talks, the engagement of the students, and the opportunities for interaction between group leaders and students were fantastic. I believe the participation of international experts had a very positive impact on the local community. Collaborations like this between EMBO and non-European countries are truly transformative. I also would like to highlight the invitation of both senior and junior group leaders, which increases the visibility of young scientists and fosters fruitful collaborations.”

“A truly outstanding meeting. The students were very engaged and asked many good questions.”

“The program was outstanding, the meeting was well organized and the time for networking at meals and poster sessions and journal clubs was ample. It was terrific that time was allowed for all questions, and there were a lot from trainees. This meant that many sessions went longer than expected, but that was a good thing because it was driven by the need to give more time to scientific discussion.”
